# Mechanisms Underlying the Anti-Aging and Anti-Tumor Effects of Lithocholic Bile Acid

**DOI:** 10.3390/ijms150916522

**Published:** 2014-09-18

**Authors:** Anthony Arlia-Ciommo, Amanda Piano, Veronika Svistkova, Sadaf Mohtashami, Vladimir I. Titorenko

**Affiliations:** Department of Biology, Concordia University, 7141 Sherbrooke Street, West, SP Building, Room 501-13, Montreal, QC H4B 1R6, Canada; E-Mails: anthony.arlia@outlook.com (A.A.-C.); amandapiano91@hotmail.com (A.P.); klubnika_veronika@hotmail.com (V.S.); sadaf.mohtashami@gmail.com (S.M.)

**Keywords:** bioactive lipids, lipid metabolism, lipidomics, bile acids, aging and age-related diseases, cancer, anti-aging and anti-tumor therapeutic agents, endoplasmic reticulum, mitochondria, cell death

## Abstract

Bile acids are cholesterol-derived bioactive lipids that play essential roles in the maintenance of a heathy lifespan. These amphipathic molecules with detergent-like properties display numerous beneficial effects on various longevity- and healthspan-promoting processes in evolutionarily distant organisms. Recent studies revealed that lithocholic bile acid not only causes a considerable lifespan extension in yeast, but also exhibits a substantial cytotoxic effect in cultured cancer cells derived from different tissues and organisms. The molecular and cellular mechanisms underlying the robust anti-aging and anti-tumor effects of lithocholic acid have emerged. This review summarizes the current knowledge of these mechanisms, outlines the most important unanswered questions and suggests directions for future research.

## 1. Introduction

Aging of unicellular and multicellular eukaryotic organisms is an intricate biological phenomenon [1,2,3,4,5]. It is believed to be caused by an age-dependent, progressive dysregulation of many processes within a eukaryotic cell [6,7,8,9,10,11,12,13,14,15,16,17,18,19]. The rates, efficiencies and spatiotemporal organization of all of these cellular processes throughout the organismal lifespan are modulated by only a few nutrient- and energy-sensing signaling pathways that converge into a network; this evolutionarily conserved network integrates the insulin/insulin-like growth factor 1, AMP-activated protein kinase/target of rapamycin and cAMP/protein kinase A (cAMP/PKA) pathways [6,7,8,9,10,11,12,13,20,21,22,23,24,25,26,27,28,29,30,31,32,33,34,35]. The flow of information along the signaling network of cellular aging can be modulated by certain dietary and pharmacological interventions that can extend lifespan and/or delay the onset of various age-related physiological changes in yeast, nematodes, fruit flies, mice and primates. These interventions are known to prolong both longevity and healthspan in organisms across phyla by beneficially influencing pathologies and diseases of old age [3,4,5,6,7,8,27,28,29,30,31,32,34,35,36,37,38,39,40]. These interventions include: (1) caloric restriction (CR), a dietary regimen that limits the intake of calories without reducing the supply of amino acids, vitamins and other nutrients [3,6,7,8,9,37,38,39,40,41,42,43,44,45,46,47,48,49,50,51,52]; (2) dietary restriction (DR), a group of nutrient intake interventions that limit the supply of certain amino acids or vitamins and/or alter the balance of dietary components, but do not reduce overall food or calorie intake [3,6,38,39,40,41,53,54,55,56,57,58,59,60,61,62,63,64,65,66,67,68,69,70,71]; and (3) certain natural chemical compounds and some pharmaceutical drugs [3,6,7,8,9,31,34,35,36,37,38,39,40,72,73,74,75,76,77,78,79,80,81,82,83,84,85,86,87,88,89,90,91,92,93,94,95,96,97,98,99,100,101,102,103,104,105,106].

The molecular and cellular mechanisms underlying the robust longevity-extending and health-improving effects of CR, certain DR regimens and some pharmacological interventions have begun to emerge. These mechanisms involve several distinct, evolutionarily conserved ways of modulating the flow of information along the signaling network, which orchestrates a pro- or anti-aging cellular pattern by controlling numerous longevity-defining cellular processes [3,4,5,6,7,8,9,10,11,12,13,24,35,36,37,38,39,40,44,45,46,47,48,49,50,51,91,97,98,99]. Among these cellular processes are certain pathways of lipid metabolism and interorganellar transport [14,15,16,17,18,94,95,107,108,109,110,111,112,113,114,115,116,117,118,119,120,121,122,123,124]. Although it remains to be seen if these various pathways can play casual roles in defining longevity and/or healthspan, recent findings suggest that at least some of them can. In fact, it has been demonstrated that: (1) sphingolipid metabolism defines yeast chronological lifespan by modulating many vital cellular processes [125,126,127,128]; (2) the lipolysis of triacylglycerols (TAG), a major class of neutral lipids, defines the longevity of the nematode *Caenorhabditis elegans* by providing arachidonic fatty acid, which extends lifespan by stimulating the essential pro-longevity process of autophagy [118,129]; and (3) the concentration of long-chain fatty acids in plasma is a probable biomarker of longevity in various species of mammals [130].

**Figure 1:**
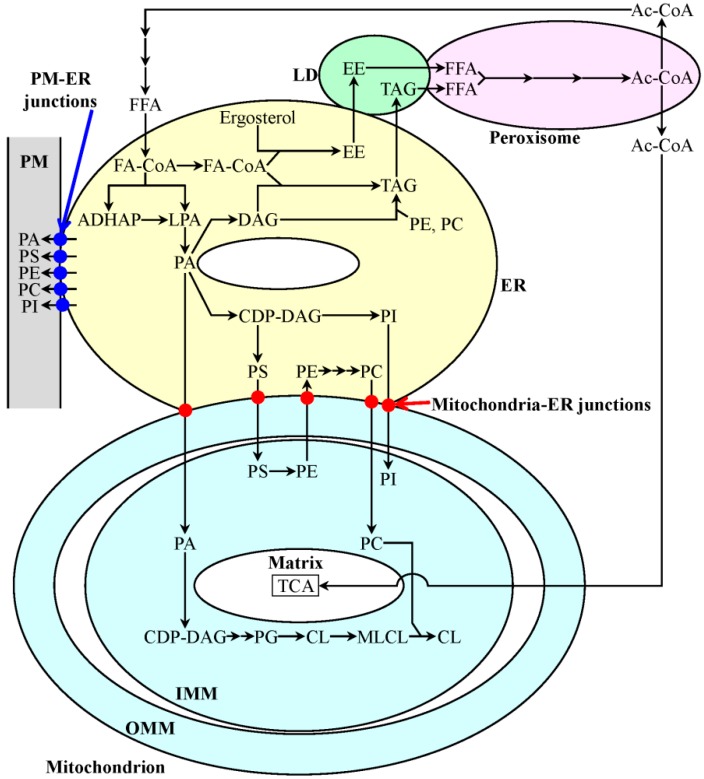
Outline of a network governing lipid metabolism and transport within the endoplasmic reticulum (ER), lipid droplets (LD), peroxisomes, mitochondria and the plasma membrane (PM). The proper functioning of this intricate network is necessary for maintaining lipid homeostasis in all of these cellular organelles and membranes. The PA, PS, PC and PI classes of phospholipids are synthesized exclusively in the ER; these are then transported to mitochondria via mitochondria-ER junctions and to the PM via PM-ER junctions. The PE and CL classes of phospholipids are formed only in the inner mitochondrial membrane (IMM); PE is then transported from mitochondria to the ER via mitochondria-ER junctions and from the ER to the PM via PM-ER junctions. The neutral lipids TAG and EE are synthesized in the ER and then deposited within LD. The lipolytic hydrolysis of TAG and EE in LD generates FFA; these then get imported and oxidized by peroxisomes. Peroxisomally produced acetyl-CoA is converted to citrate and acetyl-carnitine, whose subsequent delivery to mitochondria enables one to maintain the efficient synthesis of PE and CL in the IMM. The use of peroxisomally produced acetyl-CoA for the synthesis of FFA in the cytosol allows FFA to enter the biosynthetic pathways for phospholipids and neutral lipids in the ER. See the text for additional details. Abbreviations: Ac-CoA, acetyl-CoA; ADHAP, acyl dihydroxyacetone phosphate; CDP-DAG, cytidine diphosphate-diacylglycerol; CL, cardiolipin; EE, ergosteryl esters; FA-CoA, fatty acid-CoA; FFA, non-esterified (free) fatty acids; LPA, lysophosphatidic acid; MLCL, monolysocardiolipin; OMM, outer mitochondrial membrane; PA, phosphatidic acid; PC, phosphatidylcholine; PE, phosphatidylethanolamine; PG, phosphatidylglycerol; PI, phosphatidylinositol; PS, phosphatidylserine; TAG, triacylglycerols; WT, wild-type.

**Figure 2:**
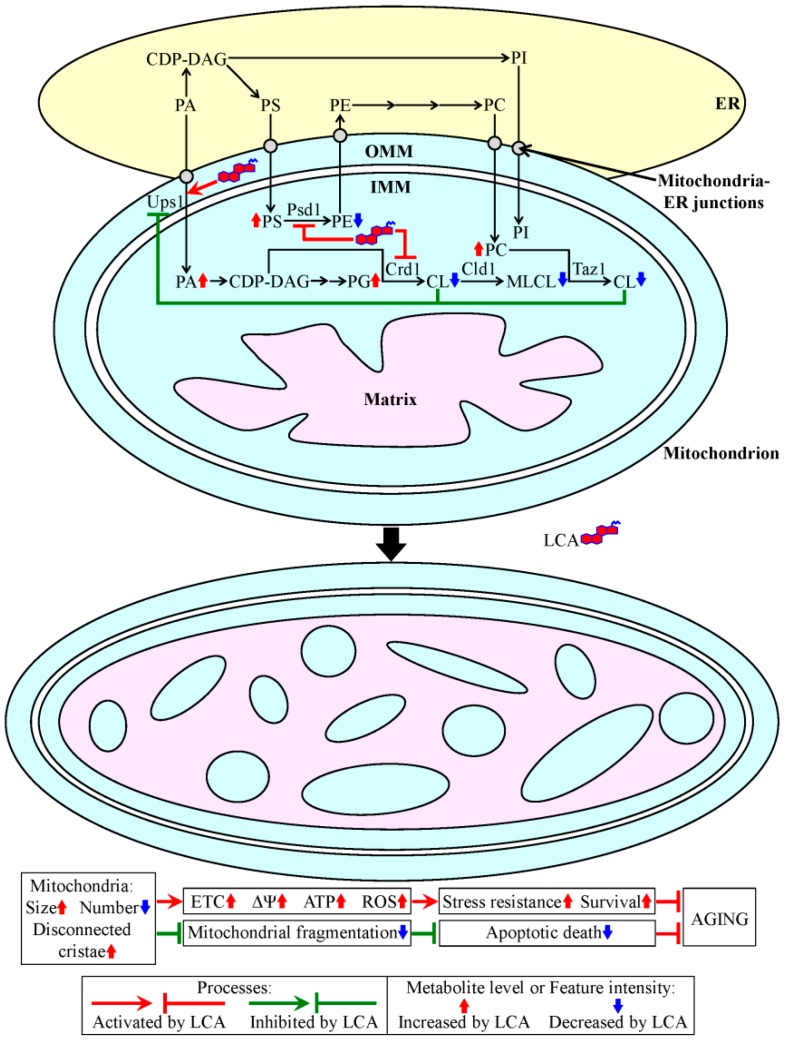
A mechanism through which lithocholic acid (LCA) prolongs the longevity of chronologically aging yeast. Exogenously added LCA enters a yeast cell, where it is sorted to mitochondria, but not to any other organelle. Mitochondria-associated LCA is located predominantly in the inner mitochondrial membrane (IMM) and also resides in the outer mitochondrial membrane (OMM). LCA drives a remodeling of the mitochondrial membrane lipidome, thereby enlarging mitochondria, reducing their number and causing a build-up within their matrix of cristae disconnected from the IMM. These major changes in mitochondrial abundance and morphology elevate mitochondrial respiration, membrane potential, ATP synthesis and reactive oxygen species (ROS) levels in chronologically “old” cells, thereby enhancing their long-term stress resistance and viability. Moreover, the LCA-elicited remodeling of the mitochondrial membrane lipidome mitigates mitochondrial fragmentation, thus slowing down an age-related form of apoptotic programmed cell death. All of these distinctive alterations in vital mitochondrial processes and features seen in yeast cells permanently exposed to exogenous LCA extend their chronological lifespan. See the text for additional details. Abbreviations: CDP-DAG, cytidine diphosphate-diacylglycerol; CL, cardiolipin; ER, endoplasmic reticulum; ETC, electron transport chain; MLCL, monolysocardiolipin; PA, phosphatidic acid; PC, phosphatidylcholine; PE, phosphatidylethanolamine; PG, phosphatidylglycerol; PI, phosphatidylinositol; PS, phosphatidylserine; ΔΨ, electrochemical membrane potential.

It needs to be emphasized that: (1) lipid metabolism and transport within a cell are governed by an intricate network of interorganellar communications integrating the endoplasmic reticulum (ER), lipid droplets (LD), peroxisomes, mitochondria and the plasma membrane (PM) [10,11,16,17,18,124,131,132,133,134,135,136,137,138,139,140] (Figure 1); (2) the proper functioning of this network is essential for maintaining lipid homeostasis in all of these cellular organelles and membranes [10,11,16,17,18,95,124,133,136,137,138,139,140] (Figure 1); and (3) the efficacy of maintaining lipid homeostasis in some or all of these cellular organelles and membranes defines the lifespan of chronologically aging yeast [10,11,16,17,18,95]. A current view of how the network integrating lipid metabolism and transport in different cellular locations maintains lipid homeostasis in various cellular organelles and membranes is summarized in a model; this model is depicted schematically in Figure 1. The model posits that: (1) after being synthesized in the ER, the phosphatidic acid (PA), phosphatidylserine (PS), phosphatidylcholine (PC) and phosphatidylinositol (PI) classes of phospholipids are transported to mitochondria via mitochondria-ER junctions and to the PM via PM-ER junctions; (2) following the synthesis of the phosphatidylethanolamine (PE) class of phospholipids in the inner mitochondrial membrane (IMM) from PS formed in the ER, PE is transported from mitochondria to the ER via mitochondria-ER junctions and subsequently from the ER to the PM via PM-ER junctions; (3) cardiolipin (CL), a signature phospholipid class of the mitochondrion, is synthesized in the IMM from PA, which is formed in the ER and then delivered to mitochondria via mitochondria-ER junctions; (4) after being synthesized in the ER, the neutral lipids triacylglycerols (TAG) and ergosteryl esters (EE) are deposited within LD; (5) the physical contact existing between peroxisomes and LD stimulates the lipolytic conversion of TAG and EE to free fatty acids, which then get imported and oxidized by peroxisomes; (6) the anaplerotic conversion of acetyl-CoA to citrate and acetyl-carnitine in peroxisomes enables the replenishment of tricarboxylic acid (TCA) cycle intermediates destined for mitochondria, thereby allowing one to maintain the efficient synthesis of PE and CL in the IMM; and (7) a pool of peroxisomally produced acetyl-CoA is also used in the cytosol for the synthesis of fatty acids, which then get imported by the ER, where they enter the biosynthetic pathways for phospholipids and neutral lipids [10,11,16,17,18,124,131,132,133,134,135,136,137,138,139,140] (Figure 1).

In a high-throughput screen for chemical compounds that can slow down aging in the yeast, *Saccharomyces cerevisiae*, by specifically targeting lipid metabolism and interorganellar transport, we identified lithocholic acid (LCA), a bile acid, as one of them [95]. Our screen revealed that several other bile acids are also longevity-extending molecules. These other bile acids were deoxycholic acid, chenodeoxycholic acid, dehydrocholic acid and hyodeoxycholic acid. All of them were shown to increase yeast chronological lifespan to a significantly lesser degree than LCA, which is the most hydrophobic bile acid species [95]. Although we found that LCA considerably delays the aging of chronologically aging yeast, this unicellular eukaryote does not synthesize LCA or any other bile acid produced and released into the environment by mammals [95,135]. In mammals, bile acids play essential roles in many processes known to be required for the maintenance of a healthy lifespan [141,142,143,144,145,146,147,148,149,150,151]; these roles are outlined in more detail below. In this review, we discuss recent progress in understanding the molecular and cellular mechanisms by which LCA, a cholesterol-derived bioactive lipid, delays chronological aging in the yeast, *S. cerevisiae*, and exhibits potent and specific anti-tumor effects in cultured cancer cells derived from different tissues and organisms.

## 2. Bile Acids Extend Healthy Lifespan in Multicellular Eukaryotic Organisms across Species

Primary bile acids in mammals are formed from the cholesterol backbone exclusively in hepatocytes of the liver, whereas secondary bile acids (including LCA) are the products of the enzymatic modification of primary bile acids by intestinal microbial flora [141,142,144]. Bile acids are cholesterol-derived amphipathic molecules with detergent-like properties that facilitate the emulsification and absorption of dietary lipids and fat-soluble vitamins in the small intestine, influence the composition and proliferation of the intestinal microbial flora, stimulate cholesterol solubilization in bile, promote bile secretion from hepatocytes into the bile canaliculi and enable the maintenance of organismal sterol homeostasis by being first formed from cholesterol and then released into the feces [141,142,143,144,145]. Moreover, bile acids are potent signaling molecules. In mammals, they specifically bind to and activate the nuclear farnesoid X receptor, the nuclear pregnane X receptor, the nuclear vitamin D receptor and the plasma membrane-bound G protein-coupled TGR5 (a protein that in humans is encoded by the *GPBAR1* gene) receptor, thus stimulating many longevity- and healthspan-promoting processes in various tissues [141,144,146,147,148,149,150,151]. These processes include mitochondrial oxidative metabolism, energy expenditure regulation, glucose metabolism and insulin sensitivity, metabolism of cholesterol and neutral lipids, maintenance of bile acid homeostasis, detoxification of xenobiotic and endobiotic toxins, growth of intestinal microbial organisms, hepatoprotection and liver regeneration and anti-inflammatory processes [141,142,143,144,148,149,150,151]. Because of the numerous beneficial effects of bile acids on longevity- and healthspan-promoting processes, they are used (or have the great potential to be used) as therapeutic agents for several age-related metabolic and neurodegenerative disorders caused by dysregulation of these processes [141,142,148,149]. For example, ursodeoxycholic acid has been used for solubilizing cholesterol gallstones, improving liver function in patients with primary biliary cirrhosis and preventing the occurrence of veno-occlusive disease in recipients of bone marrow transplants [141,142,149]. Furthermore, cholic and chenodeoxycholic bile acids have been successfully used for increasing lipid absorption and improving liver function, thereby preventing progressive liver disease in patients with inborn errors of bile acid synthesis [141,142,149]. Moreover, several natural and synthetic bile-acid classes of agonists of the nuclear farnesoid X receptor or TGR5 receptor are currently undergoing clinical trials for their potential use in the treatment of type 2 diabetes, non-alcoholic fatty liver disease, metabolic syndrome or primary biliary cirrhosis [141,142,148,149]. It needs to be emphasized that none of the above bile acids has been reported to exhibit potent anti-aging or anti-tumor effects similar to the ones we found to be characteristic of LCA.

Importantly, recent findings suggest that bile acids may extend lifespan in mice by stimulating age-related hormetic responses and, thus, by acting as endobiotic regulators that slow down the aging process [152,153,154,155]. In fact, the levels of several bile acids have been found to be increased in the long-lived Ghrhr^lit/lit^ mouse, which exhibits attenuated signaling through the pro-aging insulin/insulin-like growth factor 1 pathway, due to considerably reduced circulating levels of insulin-like growth factor 1 [152,153]. Moreover, the administration of cholic bile acid to the food of wild-type mice has been shown to stimulate the transcription of many xenobiotic detoxification genes [152,153]. Furthermore, bile acid-like dafachronic acids in the nematode, *C. elegans*, are known to act as cell non-autonomous molecular signals that extend organismal longevity by activating a transcriptional program orchestrated by the DAF-12/DAF-16 signaling cascade [115,156,157,158,159,160,161,162,163]. In sum, these findings strongly suggest that bile acids in animals may function as signaling molecules that stimulate a distinct set of vital longevity- and healthspan-promoting processes. Because we found that LCA considerably (and some other bile acids to a lesser degree) increases the chronological lifespan of yeast [95], it is conceivable that the mechanisms by which bile acids extend healthy lifespan have been conserved during the course of evolution.

## 3. A Mechanism Underlying the Longevity-Extending Effect of LCA (Lithocholic Acid) in Chronologically Aging Yeast

Our recent studies uncovered a mechanism through which LCA prolongs the longevity of chronologically aging yeast [95,135,164,165]. With the help of subcellular fractionation by differential centrifugation, organelle separation by equilibrium density gradient centrifugation and subsequent mass spectrometric measurement of LCA in purified cellular organelles, we demonstrated that exogenously added LCA enters yeast cells and accumulates in mitochondria, but not in any other organelle [135,165] (Figure 2). Using subfractionation of purified mitochondria followed by mass spectrometric quantitation of LCA in different mitochondrial subcompartments, we revealed that confined to the mitochondria, LCA resides mainly in the IMM; a smaller portion of this bile acid also associates with the outer mitochondrial membrane (OMM) [135,165] (Figure 2). Our mass spectrometric analyses of mitochondrial membrane lipidomes provided evidence that the pools of LCA confined to the IMM and OMM alter the phospholipid composition of mitochondrial membranes. Specifically, LCA elicits a rise in the relative level of PS and a decline in the relative level of PE within mitochondrial membranes; one could assume that LCA may cause these changes by decelerating the Psd1-dependent reaction, leading to the conversion of PS to PE [135,165] (Figure 2). Furthermore, LCA causes a rise in the relative level of PG and a decline in the relative level of CL within mitochondrial membranes; it is conceivable that LCA may trigger these changes by slowing down the Crd1-dependent reaction resulting in the synthesis of CL from PG [135,165] (Figure 2). Moreover, LCA increases the relative level of PC, as well as reduces the relative levels of both CL and monolysocardiolipin (MLCL) within mitochondrial membranes; one could envisage that LCA may elicit these changes by reducing the availability of newly synthesized CL for the Cld1- and Taz1-driven reactions that enable a PC-dependent remodeling of the acyl chains of CL [135,165] (Figure 2). In addition, LCA was found to increase the relative level of PA within mitochondrial membranes [135,165] (Figure 2). One could assume that the observed LCA-driven reduction in the relative level of CL within the IMM may cause such an effect by mitigating a CL-dependent inhibition of PA translocation from the OMM to the IMM; this translocation is known to be promoted by the Ups1 protein, which shuttles PA between the two mitochondrial membranes [134,136,140] (Figure 2). The resulting acceleration of PA transport from the OMM to the IMM, in synergy with the LCA-stimulated movement of PA from the ER to the OMM via mitochondria-ER junctions, may elicit the observed rise in the relative level of PA within mitochondrial membranes [135,165] (Figure 2).

The observed remodeling of the mitochondrial membrane lipidome in yeast cells permanently exposed to LCA progresses with their chronological age and triggers major age-related changes in mitochondrial abundance and morphology, including: (1) an expansion of both mitochondrial membranes, which leads to a considerable enlargement of mitochondria; (2) a shift in the balance between the opposing processes of mitochondrial fission and fusion towards fusion, which causes a substantial decline in mitochondrial number; (3) a significant decrease in the fraction of mitochondria with cristae that extend from the inner boundary membrane; and (4) a massive accumulation within the mitochondrial matrix of cristae disconnected from the inner boundary membrane [135,165] (Figure 2).

In synergy, the major changes triggered by LCA in the mitochondrial membrane lipidome and the ensuing vast changes in mitochondrial morphology elicit a distinct set of alterations in the age-related chronology of several mitochondrial processes; these vital mitochondrial processes include respiration, the preservation of electrochemical membrane potential, the synthesis of ATP and the maintenance of reactive oxygen species (ROS) homeostasis [135,165] (Figure 2). Because a permanent exposure of yeast to LCA stimulates all of these mitochondrial processes in chronologically “old” cells, they exhibit higher long-term stress resistance and viability than yeast cells cultured without LCA [135,165] (Figure 2). Moreover, a shift is elicited by LCA in the balance between the opposing processes of mitochondrial fission and fusion towards fusion attenuates mitochondrial fragmentation, thus slowing down the release of pro-apoptotic proteins from mitochondria and decelerating an age-related form of apoptotic programmed cell death [135,164,165] (Figure 2). By promoting the long-term stress resistance and viability of chronologically aging yeast cells and by slowing down their age-related apoptotic death, the permanent exposure of these cells to LCA extends their longevity [135,164,165] (Figure 2).

## 4. A Hypothesis: The Mitochondria-Centered Mechanism by Which LCA Prolongs Longevity Could Be Integrated into a Network of Interorganellar Communications Underlying Cellular Aging

As discussed in the Introduction, the homeostasis of the cellular lipidome in yeast is maintained via an intricate network of interorganellar communications; this network orchestrates lipid metabolism and transport within the ER, LD, peroxisomes, mitochondria and the PM [10,11,16,17,18,124,131,132,133,134,135,136,137,138,139,140] (Figure 1). We hypothesize that the mechanism centered on the mitochondria through which LCA extends yeast chronological lifespan [95,135,164,165] (Figure 2) could converge into the network of interorganellar communications orchestrating lipid dynamics within the ER, LD, peroxisomes, mitochondria and the PM. Our hypothesis posits that the observed LCA-elicited changes in mitochondrial membrane lipidome [135,165] (Figure 2) trigger age-related alterations in the lipidomes of all other cellular organelles and membranes integrated into this network of interorganellar communication. Such age-related alterations in the lipidomes of the ER, LD, peroxisomes, mitochondria and the PM are known to define yeast chronological lifespan by modulating the flow of interorganellar information, which is essential for establishing a pro- or anti-aging cellular pattern [10,11,14,15,16,17,18,95,135,138]. It is conceivable therefore that the mitochondria-centered mechanism by which LCA prolongs yeast longevity (Figure 2) is dynamically integrated into a network of interorganellar communications underlying cellular aging in yeast; the term “an endomembrane system that governs cellular aging” has been coined to reflect the essential role of crosstalk between different intracellular compartments in regulating cellular aging [10,11,18]. One could envision the existence of the following two ways for such an integration: (1) the observed remodeling of the mitochondrial membrane lipidome in yeast cells permanently exposed to exogenous LCA may cause an age-related remodeling of lipid metabolism and transport in the ER, LD and peroxisomes, thereby postponing a recently discovered age-related mode of programmed cell death called “liponecrosis” [165]; and (2) such LCA-elicited remodeling of the mitochondrial membrane lipidome may also trigger an age-related remodeling of the central metabolism in the cytosol, thus altering the coordinated metabolite flow within glycolytic and non-glycolytic pathways of carbohydrate metabolism known to define yeast longevity by modulating a distinct set of vital cellular processes [11].

## 5. A Mechanism Underlying an Anti-Tumor Effect of LCA in Cultured Human Cancer Cells

Incidence rates of many types of cancer are known to increase with age; therefore, cancer is considered as one of the diseases associated with aging [3,38,166,167]. Significant progress has been made in our understanding of the intricate relationship that exists between the convergent and divergent mechanisms underlying aging and cancer [5,167,168,169,170,171,172,173,174,175,176,177,178,179]. Because our studies revealed that LCA slows down cellular aging in yeast by modulating several cellular processes known for their essential roles in such mechanisms [95,135,164,165], we recently investigated how this bile acid affects cultured cancer cells derived from different tissues and organisms [180,181].

**Figure 3:**
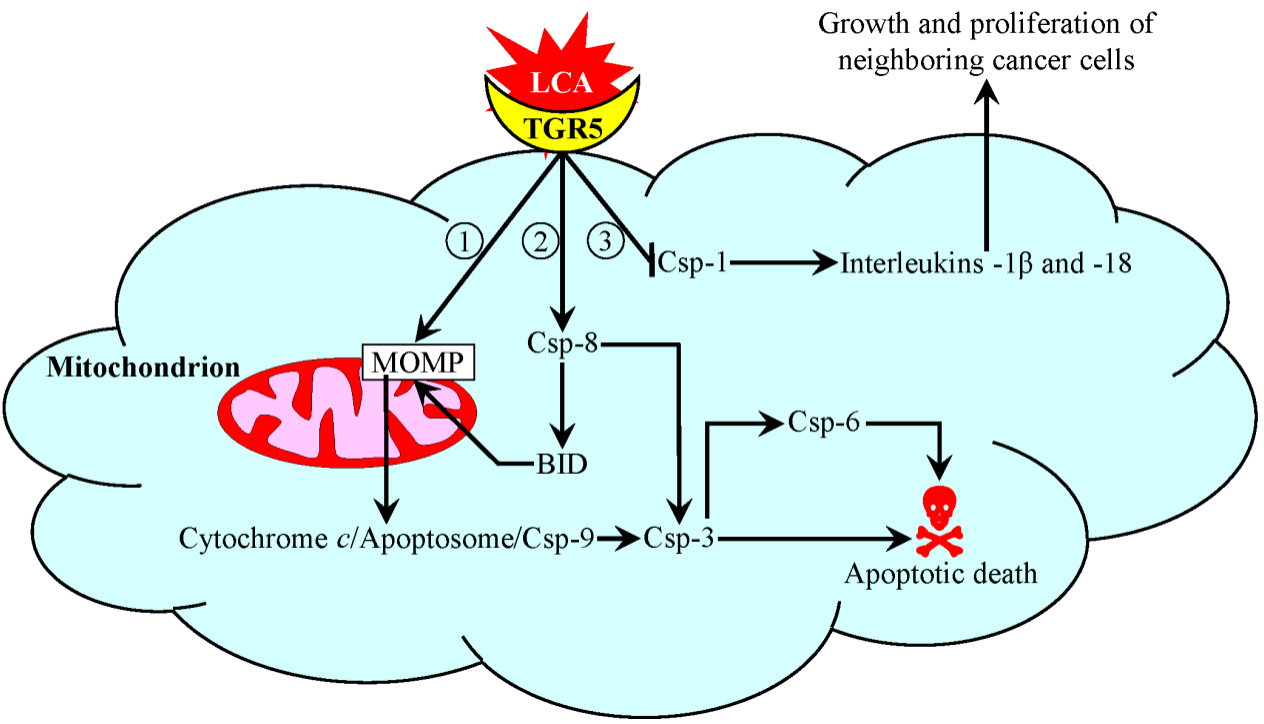
A mechanism underlying an anti-tumor effect of lithocholic acid (LCA) in cultured human neuroblastoma cells. LCA is the most potent natural agonist of TGR5, a plasma membrane-bound G protein-coupled receptor. LCA binding to TGR5 on the cell surface compromises viability and/or proliferation of human neuroblastoma cells by triggering three different pathways. The first pathway is initiated when LCA-stimulated TGR5 activates the cAMP/PKA signaling cascade. The ensuing specific changes in mitochondrial redox processes and morphology cause an activation of mitochondrial outer membrane permeabilization (MOMP), thus triggering the intrinsic (mitochondrial) pathway of apoptotic death. The second pathway leads to activation of the initiator caspase-8. Activated caspase-8 cleaves and stimulates both the executioner caspase-3 and BID (BH3-interacting domain death agonist), thus eliciting both the extrinsic (death receptor) and intrinsic (mitochondrial) pathways of apoptotic death, respectively. The third pathway operates via an inhibition of the inflammatory caspase-1, thus slowing down the processing and secretion of the cytokines interleukin-1β and interleukin-18 and, ultimately, attenuating the growth and proliferation of neighboring neuroblastoma cells. See the text for additional details. Abbreviations: Csp-1, -3, -6, -8 and -9, caspases-1, -3, -6, -8 and -9.

We found that, at concentrations that are not toxic to cultured non-cancerous cells, LCA kills cultured human neuroblastoma, breast cancer and prostate cancer cells, as well as cultured rat glioma cells [180,181]. Thus, LCA exhibits a potent and selective anti-tumor effect in cultured cancer cells that originate from diverse tissues and organisms. These studies uncovered a mechanism underlying such an anti-tumor effect of LCA in cultured human neuroblastoma cells [180] (Figure 3); a similar mechanism is responsible for the anti-tumor action of LCA in cultured human prostate cancer cells [181]. In this mechanism, LCA does not enter human neuroblastoma cells. It interacts with the plasma membrane-bound protein TGR5 on the cell surface, thereby stimulating this G protein-coupled receptor of LCA [180] (Figure 3). Such stimulation of TGR5 by LCA, its most potent natural agonist [141,149,182,183], triggers three different pathways that compromise viability and/or proliferation of human neuroblastoma cells. First, LCA-stimulated TGR5 activates the cAMP/PKA signaling pathway, thereby altering redox processes in mitochondria and mitochondrial morphology [141,143,149,184,185] (Figure 3). The ensuing activation of mitochondrial outer membrane permeabilization (MOMP) initiates the intrinsic (mitochondrial) pathway of apoptotic death by eliciting a cascade of sequential events that include the fragmentation of mitochondria, the release of cytochrome *c* from the mitochondrial intermembrane space into the cytosol, the formation of the apoptosome, the activation of the initiator caspase-9, caspase-9-dependent proteolytic activation of the executioner caspase-3, caspase-3-driven proteolytic processing and activation of the executioner caspase-6 and, ultimately, cell demolition by the executioner caspases through proteolytic cleavage of their numerous protein substrates [180] (Figure 3). Second, LCA-stimulated TGR5 also initiates the extrinsic (death receptor) pathway of apoptosis, which leads to activation of the initiator caspase-8 [180] (Figure 3); a mechanism underlying such activation remains to be characterized. The active form of caspase-8 not only proteolytically stimulates the executioner caspase-3, but can also cleave and activate the BH3-only protein, BID (BH3 interacting-domain death agonist), thus causing MOMP and triggering the intrinsic pathway of apoptosis [186,187]. Third, LCA-stimulated TGR5 reduces the activity of the inflammatory caspase-1 via a currently unknown mechanism [180] (Figure 3). This caspase is involved in the processing and secretion of interleukin-1β and interleukin-18 [188,189,190,191], two cytokines known for their essential roles in stimulating cell growth and proliferation [192,193,194]. It is conceivable therefore that the observed inhibition of the inflammatory caspase-1 in human neuroblastoma cells treated with LCA may attenuate the growth and proliferation of neighboring cancer cells, thereby contributing to the anti-tumor effect of LCA in these cells [180] (Figure 3).

## 6. Conclusions and Future Perspectives

Recent studies revealed that LCA, a bile acid, not only extends the longevity of chronologically aging yeast, but also compromises the viability and proliferation of cultured cancer cells derived from different tissues and organisms. The molecular and cellular mechanisms underlying the robust anti-aging and anti-tumor effects of this cholesterol-derived bioactive lipid have emerged. Despite significant progress in the understanding of these mechanisms, we are still lacking answers to the following important questions.

Do the LCA-driven alterations in the mitochondrial membrane lipidome seen in yeast cells [135,165] (Figure 2) elicit any changes in the membrane lipidomes of other cellular organelles and membranes known to be integrated into a network of interorganellar communication that underlies cellular aging [10,11,16,17,18,95,124,131,132,133,134,135,136,137,138,139,140] (Figure 1)? If so, how do these changes in the lipidomes of the ER, LD, peroxisomes, mitochondria and/or the PM in yeast cells permanently exposed to LCA contribute to the “liponecrotic” mode [165] of their age-related programmed cell death?

Does the remodeling of the mitochondrial membrane lipidome driven by LCA in chronologically aging yeast [135,165] (Figure 2) alter the coordinated metabolite flow within certain pathways of the central metabolism in the cytosol? If so, how do these alterations in the metabolome of yeast cells treated with LCA impact several longevity-defined cellular processes [11] that are known to be modulated by the metabolite flow within glycolytic and non-glycolytic pathways of carbohydrate metabolism?

How does LCA binding to TGR5 on the surface of human neuroblastoma cells activate the initiator caspase-8 [180] (Figure 3)? What is the mechanism underlying the ability of LCA-stimulated TGR5 to inhibit the inflammatory caspase-1 in these cancer cells [180] (Figure 3)? How does such LCA-driven inhibition of caspase-1 impact the growth and proliferation of human neuroblastoma cells?

We shall have to answer these important questions if we want to understand the inherent complexity of mechanisms through which LCA and other bioactive lipids influence cellular aging, define organismal longevity and impact diseases of old age.
